# Supplementary material for: Nuclear energy transition and CO2 emissions nexus in 28 nuclear electricity-producing countries with different income levels
Source: PeerJ. 2022 Jul 25;10:e13780. doi: 10.7717/peerj.13780 (PMC9332342; doi:10.7717/peerj.13780)
Supplement: Supplemental Information 2 [file peerj-10-13780-s002.docx]

**APPENDIX**

List of countries.

| **Income group** | **Countries** |
| --- | --- |
| High income | Canada |
|  | Czech Republic |
|  | Finland |
|  | France |
|  | Germany |
|  | Hungary |
|  | Netherlands |
|  | Slovakia |
|  | Slovenia |
|  | South Korea |
|  | Spain |
|  | Sweden |
|  | Switzerland |
|  | United Kingdom |
|  | US |
|  | Belgium |
|  | Japan |
| Upper middle income | Argentina |
|  | Brazil |
|  | Bulgaria |
|  | China |
|  | Mexico |
|  | Romania |
|  | Russian Federation |
|  | South Africa |
| Lower middle income | India |
|  | Pakistan |
|  | Ukraine |
